# Retraction: TAB3 O-GlcNAcylation promotes metastasis of triple negative breast cancer

**DOI:** 10.18632/oncotarget.28895

**Published:** 2026-10-16

**Authors:** Tao Tao, Zhixian He, Zhiming Shao, Haojie Lu

**Affiliations:** ^1^Shanghai Cancer Center and Institutes of Biomedical Sciences, Fudan University, Shanghai 200032, P.R. China; ^2^Department of Chemistry, Fudan University, Shanghai 200433, P.R. China; ^3^Department of General Surgery, Affiliated Hospital of Nantong University, Nantong 226001, P.R. China; ^4^Key Laboratory of Glycoconjugates Research Ministry of Public Health, Fudan University, Shanghai 200032, P.R. China

**This article has been retracted:** Oncotarget has concluded its investigation and identified the following issues:

- Figure 1A: The GAPDH western blot for MDA-MB-468 cells overlaps with the GAPDH blot for MDA-MB-231 cells; both overlap with GAPDH blots in Figure 1D and Figure 2B–2D (represented as GST-TAB3, Flag-TAB3, and TAB3 blots, respectively).
- Figure 1A: The GAPDH western blot for MDA-MB-231 is a duplicate of a previously published GAPDH blot in Figure 3B of article [1]. Additionally, Figure 2D of [1], which shares the same first author, contains a GAPDH blot image that was also reproduced as Flag-Tab3 in Supplementary Figure 4A.
- Figure 2C: The western blot for GST-TAB3 is a duplicate of the Figure 2D GST-TAB3 blot.
- Figure 2F: The western blots for OGT and Flag-Tab3 overlap with the Figure 3C blots for p-IkBa and NF-kB p65, respectively.
- Figure 3B: The GAPDH western blot overlaps with the NF-kB p65 blot from Figure 3C and the Figure 2F Flag-Tab3 bands.
- Figure 3E: The western blot for p38 overlaps with Flag-Tab3 bands (Figure 2F), GAPDH bands (Figure 3B) and NF-kB p65 bands (Figure 3C).
- Figure 4B: The western blots for TAK1 and TRAF6 overlap with the blots for p-TAB3 (Figure 4A) and CTD 110.6 (Figure 4C), respectively.
- Figure 4D: The pSer408-TAB3 blot was reproduced in the Figure 4D SUMO-1 blot of article [2].
- Figure 5D: The tumor images for the control and WT TAB3 groups were found in an unrelated 2014 article [3] in Figure 2A SPC-A-1 group.
- Supplementary Figures 2, 3 and 5 have multiple image duplications.

Oncotarget contacted the authors and found that the original data is unavailable. The corresponding author, Dr. Haojie Lu, confirmed multiple instances of inappropriate image similarities within the paper and between this paper and other published works. These issues compromise the reliability of the reported findings. Consequently, the Editorial decision has been made to retract this article. All authors have agreed to retract the article and apologize for any inconvenience caused.

Original article: Oncotarget. 2016; 7:22807–22818. 22807-22818. https://doi.org/10.18632/oncotarget.8182
